# Natural Resin-Based Anticorrosive Coatings from Rosin and *Croton lechleri* for Metallic Surface Protection

**DOI:** 10.3390/polym18182240

**Published:** 2026-09-15

**Authors:** Clara Isabel Ruiz-Sánchez, Priscila Anahi Suntaxi-Suntasig, Caterine Donoso, Carlos Andrés Navas-Cárdenas

**Affiliations:** Departamento de Ciencias de la Energía y Mecánica, Universidad de las Fuerzas Armadas ESPE, Av. General Rumiñahui s/n y Ambato, Sangolquí 171103, Ecuador

**Keywords:** rosin, dragon’s blood, *Croton lechleri*, anticorrosive coating

## Abstract

Corrosion of metallic materials in aggressive environments remains a major challenge affecting the durability of industrial structures and equipment. In this context, protective coatings based on natural materials have attracted increasing attention as sustainable alternatives to conventional anticorrosive systems. This study evaluated the physicochemical, thermal, and anticorrosive properties of rosin-based coatings modified with Dragon’s Blood (*Croton lechleri*). Formulations containing 0.5 wt.% and 1.0 wt.% of the natural resin were applied to carbon steel and aluminum substrates. The developed coatings were characterized by Fourier-transform infrared spectroscopy (FTIR), thermogravimetric analysis (TGA), and differential scanning calorimetry (DSC), while their anticorrosive performance was evaluated using an accelerated direct-current corrosion test in seawater for 60 min and seawater immersion tests for 30 days. Mass loss measurements, together with ANOVA and Tukey’s multiple comparison tests, were used to compare the protective performance of the different formulations. FTIR analysis suggested that the incorporation of Dragon’s Blood did not significantly modify the chemical structure of the rosin matrix, whereas TGA and DSC confirmed adequate thermal stability of the developed coatings. The formulation containing 0.5 wt.% Dragon’s Blood consistently exhibited the lowest mass loss for both carbon steel and aluminum, corresponding to reductions of approximately 55.1% and 73.2%, respectively, compared with the uncoated controls after 30 days of seawater immersion. Both the 30-day seawater immersion test and the accelerated direct-current corrosion test consistently identified the formulation containing 0.5 wt.% Dragon’s Blood as the most effective coating system. These findings demonstrate that Dragon’s Blood is a promising sustainable bio-based additive capable of enhancing the barrier properties and anticorrosive performance of rosin-based coatings for metallic substrates exposed to marine environments.

## 1. Introduction

Corrosion of metallic materials is one of the most critical challenges that affect the industrial infrastructure due to the substantial economic losses, operational risks, and environmental impacts associated with the degradation of metallic components [1]. This electrochemical process progressively deteriorates metal-based materials through their interaction with environmental factors such as moisture, oxygen, and aggressive ionic species. Corrosion significantly increases maintenance costs and reduces the service life of equipment and structures in sectors including the oil and gas, chemical, marine, transportation, and construction industries.

A major objective in materials engineering and corrosion science has been focused in developing efficient and environmentally sustainable strategies to protect metallic surfaces [2]. Among the various corrosion protection strategies, organic protective coatings have been widely recognized as one of the most effective and economically viable approaches [3]. These coatings primarily work as physical barriers that isolate the metallic substrate from corrosive environments, thereby limiting the penetration of water, oxygen, and aggressive ions [4]. On the other hand, conventional protective coatings are commonly based on petroleum-derived synthetic polymers due to their excellent mechanical properties, chemical resistance, and long-term durability. However, their dependence on non-renewable resources and the potential release of hazardous substances during production and disposal have triggered environmental concerns, encouraging the search for greener alternatives [5].

In recent years, bio-based polymers and natural resins have attracted considerable attention as sustainable materials for anticorrosive coatings. Their renewable origin, biodegradability, relatively low toxicity, and chemical functionality make them promising candidates for replacing conventional synthetic materials [6]. In addition to their environmental advantages, many natural polymers contain functional groups capable of interacting with metallic surfaces, contributing to the formation of protective films that reduce the diffusion of corrosive species and improve barrier performance.

Rosin has emerged as one of the most promising renewable materials for protective coatings. This natural resin is obtained as the solid fraction remaining after the distillation of oleoresin from *Pinus* species and consists predominantly of abietane- and pimarane-type resin acids, particularly abietic acid derivatives [7]. Its hydrophobic character, excellent film-forming ability, biodegradability, and compatibility with other natural and synthetic components have led to its extensive use in varnishes, adhesives, inks, and protective coatings [8]. Previous studies have demonstrated that rosin-based formulations can provide effective corrosion protection, particularly when combined with functional additives that improve adhesion, flexibility, moisture resistance, and coating integrity [9].

Another natural resin with promising potential is Dragon’s Blood, obtained mainly from Amazonian species of the genus *Croton*, particularly *Croton lechleri*. This resin is characterized by a complex chemical composition rich in phenolic compounds, proanthocyanidins, diterpenes, and other bioactive secondary metabolites that exhibit remarkable antioxidant properties [10]. Owing to these characteristics, Dragon’s Blood has traditionally been employed in medicinal applications, while recent studies have suggested its potential use in advanced functional materials.

The antioxidant activity of Dragon’s Blood is mainly attributed to its phenolic constituents, which may contribute to delaying oxidative reactions involved in corrosion processes. Furthermore, its resinous nature may promote the formation of continuous protective films that enhance the barrier properties of polymeric coatings by reducing the penetration of moisture and oxygen [11]. Nevertheless, the incorporation of natural plant resins into protective coatings remains relatively unexplored, particularly regarding the influence of resin concentration on coating performance and long-term corrosion resistance.

Despite the growing interest in sustainable anticorrosive materials, research on coatings formulated exclusively from renewable natural resins remains limited, highlighting the need for more comprehensive investigations in this field. In particular, there is limited information regarding the synergistic use of rosin and Dragon’s Blood resin, especially concerning the effect of Dragon’s Blood concentration on the physicochemical, thermal, and anticorrosive properties of these bio-based coatings. Addressing this knowledge gap may contribute to the development of environmentally friendly coating systems capable of reducing the dependence on petroleum-derived materials while maintaining satisfactory protective performance.

Recent studies have demonstrated that *Croton lechleri* (Dragon’s Blood) resin has significant potential as a green corrosion inhibitor due to the presence of phenolic compounds, proanthocyanidins, and other secondary metabolites capable of adsorbing onto metallic surfaces and reducing corrosion processes. In this regard, Cevallos-Morillo et al. reported that *Croton lechleri* extracts effectively inhibited the corrosion of admiralty brass in hydrochloric acid by forming a protective film on the metal surface [12]. These findings highlight the potential of this natural resin as a sustainable alternative to conventional corrosion inhibitors. However, most previous studies have focused on the use of *Croton lechleri* extracts as corrosion inhibitors in solution [13,14], whereas limited information is available on their incorporation as functional additives into natural polymeric matrices for the development of anticorrosive coatings intended for marine environments.

Accordingly, this study focuses on the development and evaluation of bio-based anticorrosive coatings formulated from rosin and Dragon’s Blood resin, as well as the influence of Dragon’s Blood concentration on their physicochemical, thermal, and corrosion-protection performance when applied to carbon steel and aluminum substrates. The coatings were characterized by Fourier-transform infrared spectroscopy (FTIR), thermogravimetric analysis (TGA), and differential scanning calorimetry (DSC), whereas their anticorrosive performance was evaluated using a 30-day gravimetric seawater immersion test and an accelerated direct-current corrosion test. By exploring the synergistic use of two renewable natural resins, this work contributes to the development of more sustainable protective coating systems with potential applications for metallic surface protection.

## 2. Materials and Methods

### 2.1. Preparation of Metallic Substrates

Forty-eight metallic substrates were prepared: 24 aluminum plates and 24 carbon steel plates. Specimens with a surface area of 21.25 cm^2^ (8.5 cm × 2.5 cm) were used for this study. Prior to coating application, the substrates were wet-sanded using 120-grit aluminum oxide abrasive paper (T277 ALOX) to obtain a homogeneous surface finish and promote coating adhesion [3]. The surfaces were subsequently degreased by wiping them with a lint-free cloth soaked in acetone to remove grease, dust, and other contaminants that could interfere with coating adhesion. After cleaning, each substrate was placed on aluminum foil inside a desiccator until coating application to minimize surface contamination.

The experimental design included four treatment conditions: an uncoated control (blank), a rosin coating, a rosin coating containing 0.5 wt.% Dragon’s Blood resin, and a rosin coating containing 1.0 wt.% Dragon’s Blood resin. These treatments were evaluated using two independent corrosion assessment methods: a 30-day seawater immersion test and an accelerated direct-current corrosion test. For the immersion test, the coated face of each specimen (21.25 cm^2^) was exposed to the corrosive medium, while the opposite face remained uncoated to allow visual comparison of the corrosion process. In contrast, during the accelerated direct-current corrosion test, both faces of each specimen were coated. Due to the experimental configuration, an approximately 0.5 cm section at one end of each specimen remained outside the electrolyte to allow electrical connection to the power supply. Consequently, this portion did not participate in the corrosion process and was not included in the exposed area. Three specimens were prepared for each treatment, substrate, and corrosion test. Consequently, twelve specimens per material (four treatments × three replicates) were evaluated in each corrosion test, resulting in a total of forty-eight metallic substrates used throughout the study, as summarized in Table 1.

### 2.2. Preparation of Natural Coatings

Rosin was used as the natural film-forming resin, whereas Dragon’s Blood (*Croton lechleri*) resin served as the bio-based functional additive. Commercial rosin was purchased from Laboratorio Cevallos (Guayaquil-Ecuador; Lot No. 7190055; manufacturing date: August 2023; expiration date: July 2028). Dragon’s Blood resin was obtained as a handcrafted liquid resin from an artisanal source and used without further purification. The selection of these materials was based on their renewable origin, biodegradability, and compatibility with sustainable coating formulations aimed at partially replacing conventional synthetic coatings. Owing to its high content of resin acids, rosin exhibits excellent film-forming ability and good adhesion to metallic substrates, making it a promising material for environmentally friendly protective coatings [4].

Prior to formulation, the solid rosin was manually crushed into small fragments to facilitate dissolution. Subsequently, 10 g of rosin were mixed with 50 mL of 96% ethanol and heated on a hot plate at 60 °C until a homogeneous solution free of visible solid particles was obtained [5]. During heating, the beaker was covered with a lid to minimize solvent evaporation. The temperature was carefully maintained at 60 °C, well below the boiling point of ethanol (78.4 °C), to avoid boiling throughout the dissolution process and promote uniform resin dissolution. All formulations were prepared under identical experimental conditions using the same initial solvent volume, heating temperature, and preparation procedure to ensure experimental consistency.

Three coating formulations were prepared: pure rosin, rosin containing 0.5 wt.% Dragon’s Blood resin, and rosin containing 1.0 wt.% Dragon’s Blood resin, using 10 g of rosin as the reference for additive incorporation. The composition of each formulation is summarized in Table 2. After complete dissolution of the rosin, Dragon’s Blood resin was gradually added under constant stirring to ensure its uniform distribution throughout the resin matrix and to prevent the formation of agglomerates. The mixture was stirred using a magnetic stirrer (MSH-420, BOECO, Hamburg, Germany) set at instrument setting 4 for 3 min while maintaining the temperature at 60 °C, followed by an additional 2 min under the same conditions until a visually homogeneous mixture was obtained.

The formulations were then removed from the heating source and allowed to cool naturally to room temperature. Finally, each coating was transferred to airtight glass containers, properly labeled, and stored at room temperature until application in order to minimize solvent loss and prevent contamination [5].

### 2.3. Application and Curing of Coatings

The prepared coatings were manually applied onto the previously conditioned aluminum and carbon steel substrates using a brush to obtain a continuous and uniform coating over the metallic surface. The coating was evenly distributed to avoid excessive material accumulation and to obtain a visually homogeneous layer.

For the seawater immersion test, the coating was applied to only one side of each metallic substrate, leaving the opposite side uncoated to facilitate the visual comparison between protected and unprotected surfaces after corrosion exposure. In contrast, the specimens used for the accelerated direct-current corrosion test were coated on both sides. Due to the experimental configuration, an approximately 0.5 cm section at one end of each specimen remained outside the electrolyte to allow electrical connection to the DC power supply. Consequently, this portion did not participate in the corrosion process. The experimental configuration is described in Section 2.5.2.

A single coating layer was applied to all specimens, as the rheological properties and film-forming capacity of the formulations provided continuous and visually homogeneous surface coverage without requiring additional applications. After coating application, the specimens were left to dry at room temperature for 24 h, allowing gradual solvent evaporation and initial film formation.

Subsequently, the coated specimens underwent thermal curing in an oven at 60 °C for 2 h [8]. This treatment promoted consolidation of the resin matrix, reduced residual solvent content, and contributed to the formation of a more homogeneous coating film.

After curing, the specimens were allowed to cool naturally to room temperature and stored in a desiccator until physicochemical characterization, DC corrosion testing, and seawater immersion testing.

### 2.4. Physicochemical and Thermal Characterization of the Coatings

Fourier-transform infrared (FTIR) spectra of the dried formulations were obtained using a PerkinElmer Frontier FTIR spectrometer (PerkinElmer Inc., Waltham, MA, USA) equipped with an attenuated total reflectance (ATR) cell. Analyses were performed in the spectral range of 4000–600 cm^−1^ with a resolution of 4 cm^−1^ and 32 scans per sample. The obtained IR spectra were analyzed to identify the characteristic functional groups present in the rosin and Dragon’s Blood (*Croton lechleri*) resin formulations.

Likewise, thermogravimetric (TGA) and differential scanning calorimetry (DSC) analyses were performed for each sample.

Due to the fact that thermal analyses require dry solid samples, the liquid coating formulations were conditioned prior to (TGA) and (DSC) [9]. The formulations were spread onto wax paper and allowed to dry at room temperature until complete solvent evaporation. The resulting solid material was carefully removed and gently ground using a mortar until a fine powder suitable for thermal analysis was obtained. The powdered samples were then transferred to sealed Petri dishes and stored in a desiccator to prevent moisture adsorption prior to analysis [6].

TGA was conducted on a PerkinElmer Pyris 1 thermogravimetric analyzer (PerkinElmer Inc., Waltham, MA, USA) under an air atmosphere (20 mL min^−1^). Approximately 4.4 ± 0.2 mg of each sample was heated from 25 to 450 °C at a heating rate of 10 °C min^−1^. Meanwhile, DSC was performed using a Mettler Toledo STARe System (Mettler-Toledo AG, Greifensee, Switzerland) under a nitrogen atmosphere. Approximately 4.5 ± 0.5 mg of each sample was heated from 20 to 450 °C at a heating rate of 10 °C min^−1^.

### 2.5. Evaluation of Anticorrosive Behavior

The anticorrosive performance of the prepared coatings was evaluated by a seawater immersion test and an accelerated direct-current (DC) corrosion test [12]. These complementary methods were used to assess the protective performance of the coatings under aggressive corrosive conditions.

#### 2.5.1. Seawater Immersion Test

Seawater was selected as the corrosive medium due to its high concentration of dissolved salts and its highly aggressive nature toward metallic materials, which promotes the acceleration of corrosion processes [15]. For the corrosion tests, seawater was collected from the coastal area of Manta, Manabí Province, Ecuador. According to published oceanographic data for the Ecuadorian coastal region, seawater in this area typically exhibits a salinity ranging from 33 to 35%, pH values between 8.0 and 8.2, a chloride concentration of approximately 19–20 g/L, and a conductivity in the range of 50–60 mS/cm. These values are presented only to describe the typical physicochemical characteristics of the marine environment in the sampling area, since they were not experimentally determined for the seawater used in this study [9].

The immersion tests were performed using carbon steel and aluminum specimens coated with the different formulations described in Section 2.2, including an uncoated control [10]. Three specimens were prepared for each treatment, resulting in four experimental groups for each substrate (blank, rosin, rosin + 0.5 wt.% Dragon’s Blood, and rosin + 1.0 wt.% Dragon’s Blood).

As described in Section 2.3, only one side of each specimen was coated, while the opposite side remained uncoated to facilitate visual comparison after immersion. All specimens were prepared following the same coating application procedure and drying conditions to ensure consistent experimental conditions among treatments. The specimens were completely immersed in seawater and stored under static conditions at room temperature in covered containers to prevent external contamination [11].

The exposure period used for the immersion test for each sample was 30 days. During the immersion test, the specimens were periodically examined for the appearance of corrosion products, oxide formation, coating degradation, and possible coating detachment. After the immersion period, the specimens were rinsed with distilled water to remove residual seawater salts and loosely adhered surface deposits, allowed to dry at room temperature, and subsequently weighed to determine the mass loss.

#### 2.5.2. Accelerated DC Corrosion Test

To further evaluate the anticorrosive performance of the developed coatings, an accelerated DC corrosion test was conducted under laboratory conditions. Seawater was used as the electrolyte in a 500 mL glass beaker.

For each experiment, the coated metallic specimen (carbon steel or aluminum) was connected to the positive terminal of a DC power supply, whereas an uncoated specimen of the same metallic substrate was connected to the negative terminal and served as the auxiliary electrode. A constant potential of 3 V was applied for 60 min to accelerate the corrosion process.

Due to the configuration of the electrical connections, the coated specimens were partially immersed in the electrolyte to prevent contact between the seawater and the electrical clamps. Both faces of each specimen were coated, and the portion used for the electrical connection remained outside the electrolyte throughout the experiment. The relative position of the coated specimen and the auxiliary electrode was maintained constant during all tests to ensure comparable testing conditions.

After the test, the specimens were rinsed with distilled water, allowed to dry at room temperature, and weighed to determine the mass loss. All experiments were performed in triplicate for each coating formulation, including uncoated control specimens.

## 3. Results

### 3.1. Physicochemical and Thermal Characterization of the Coatings

The ATR-FTIR spectra of rosin-based sample exhibited the characteristic absorption bands associated with its diterpene resin acid structure. The most intense absorption band was observed in the 1690–1710 cm^−1^ region, which has been assigned to the stretching vibrations of carbonyl (C=O) groups present in resin acids. Additional absorption bands between 2850 and 2950 cm^−1^ corresponded to aliphatic C–H stretching vibrations, whereas bands in the 1200–1300 cm^−1^ region were attributed to C–O stretching vibrations. Likewise, the IR spectrum of Dragon’s Blood exhibited a broad absorption band in the 3200–3500 cm^−1^ region, characteristic of hydroxyl (–OH) groups associated with phenolic compounds [13,14].

**Figure 1:**
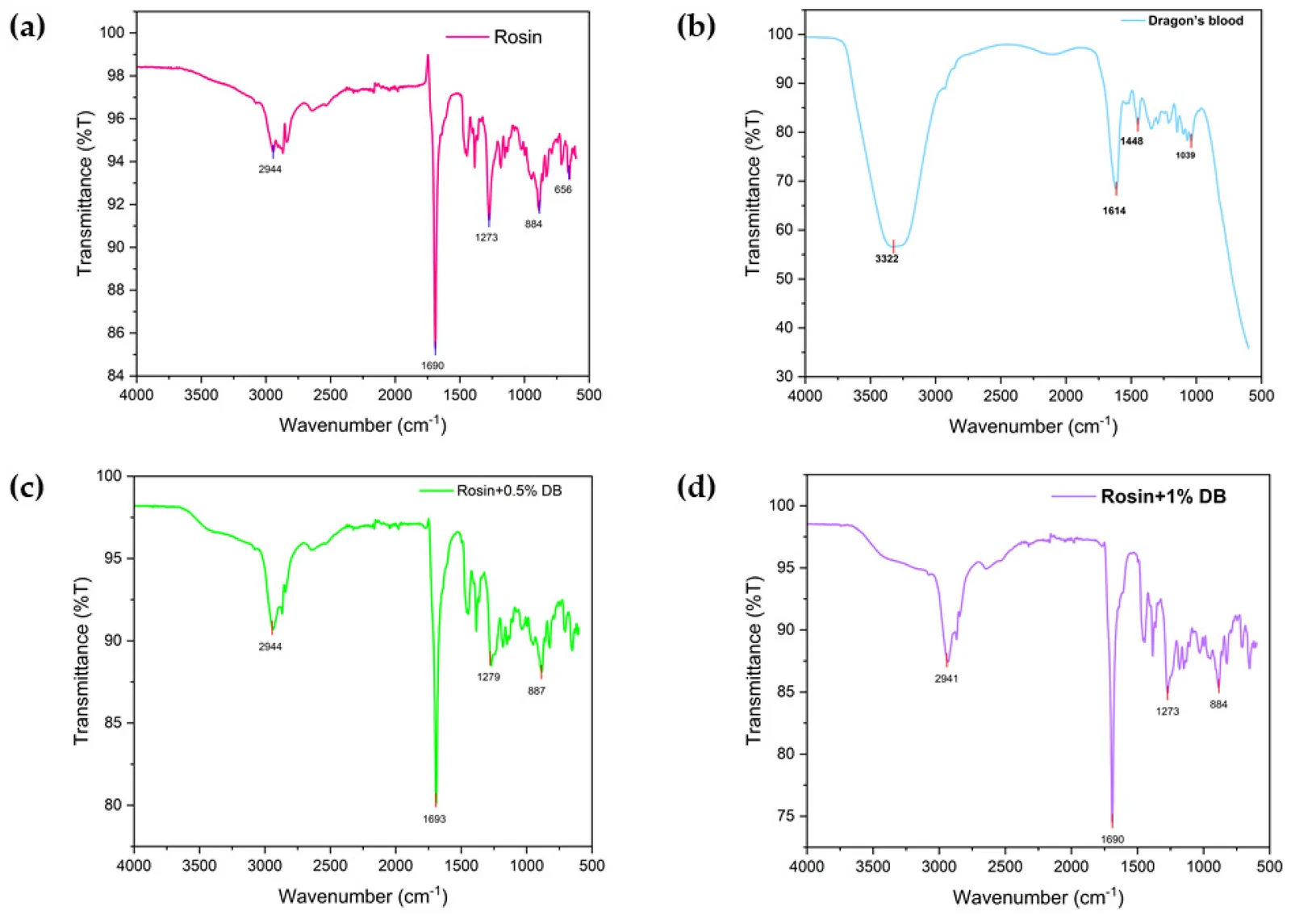
FTIR spectra of the coating components and formulations: (**a**) Rosin; (**b**) Dragon’s Blood (DB); (**c**) Rosin + 0.5 wt.% DB; and (**d**) Rosin + 1.0 wt.% DB.

Figure 1 shows the FTIR spectra of the modified formulations. The IR spectra of the modified formulations preserved the characteristic absorption bands of rosin, indicating that the incorporation of Dragon’s Blood did not significantly alter the chemical structure of the resin matrix. No new characteristic absorption bands or noticeable band shifts were observed, suggesting that no significant chemical interactions leading to the formation of new covalent bonds occurred during formulation. Instead, the interaction between both natural resins is likely governed mainly by physical interactions, including hydrogen bonding and other intermolecular forces. A slight broadening of the absorption band in the 3200–3500 cm^−1^ region was more evident with increasing Dragon’s Blood content, particularly in the formulation containing 1.0 wt.%, which is attributed to the hydroxyl-rich phenolic constituents naturally present in Dragon’s Blood resin. A similar behavior has been reported for rosin-based coatings modified with natural plant extracts, where the incorporation of bioactive additives does not substantially modify the characteristic functional groups of the resin matrix but instead promotes intermolecular interactions between the components [15,16].

**Figure 2:**
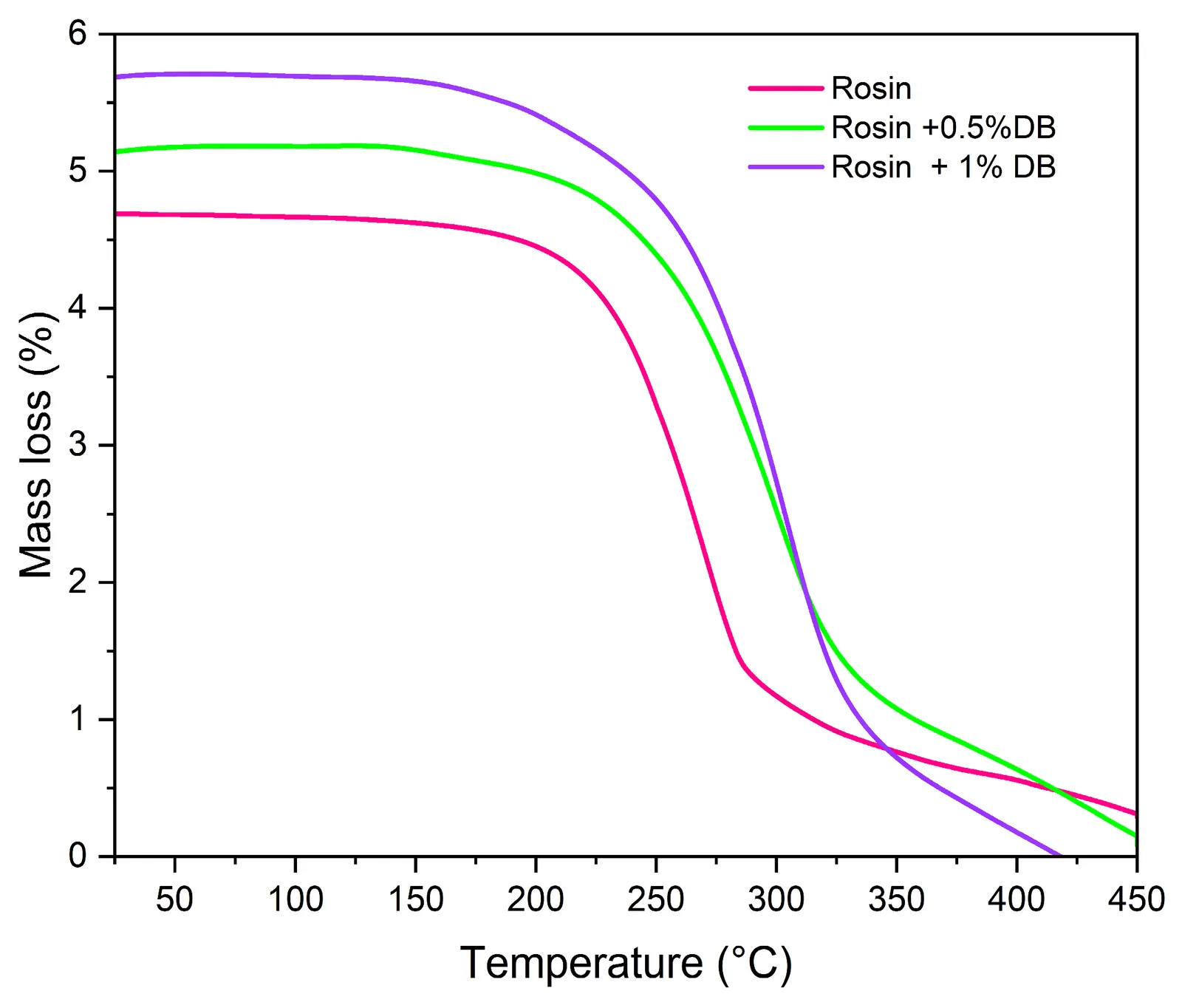
Thermogravimetric (TGA) curves of rosin-based coatings modified with different concentrations of Dragon’s Blood (Croton lechleri).

The thermogravimetric analysis (TGA) shown in Figure 2 revealed that all samples exhibited an initial mass loss between 30 and 200 °C. This mass loss can be attributed to the evaporation of residual ethanol remaining from the coating preparation process, together with the release of physically adsorbed moisture and low-molecular-weight volatile compounds naturally present in the resins.

The main degradation stage occurred between 230 and 320 °C, which corresponds to the thermal decomposition of the rosin matrix and its resin acids. The characteristic thermal degradation parameters obtained from the TGA curves are summarized in Table 3.

The incorporation of 0.5 wt.% Dragon’s Blood increased the T5% and T10% values from 199.8 and 220.4 °C to 210.3 and 234.9 °C, respectively, suggesting an improvement in the initial thermal stability of the coating. In contrast, the formulation containing 1.0 wt.% Dragon’s Blood exhibited a slightly lower T5% value (195.8 °C), although its T10% remained higher than that of pure rosin. Both modified formulations displayed a substantial increase in Tmax, from 269.6 °C for pure rosin to approximately 300 °C (299.8–299.9 °C), suggesting that the incorporation of Dragon’s Blood contributed to improved thermal stability during the main degradation stage of the resin matrix. Similar improvements in the thermal stability of rosin-based materials modified with natural bioactive additives have been reported previously, where the incorporation of plant-derived compounds promoted stronger intermolecular interactions within the resin matrix, thereby delaying the main thermal degradation stage [17,18,19].

**Figure 3:**
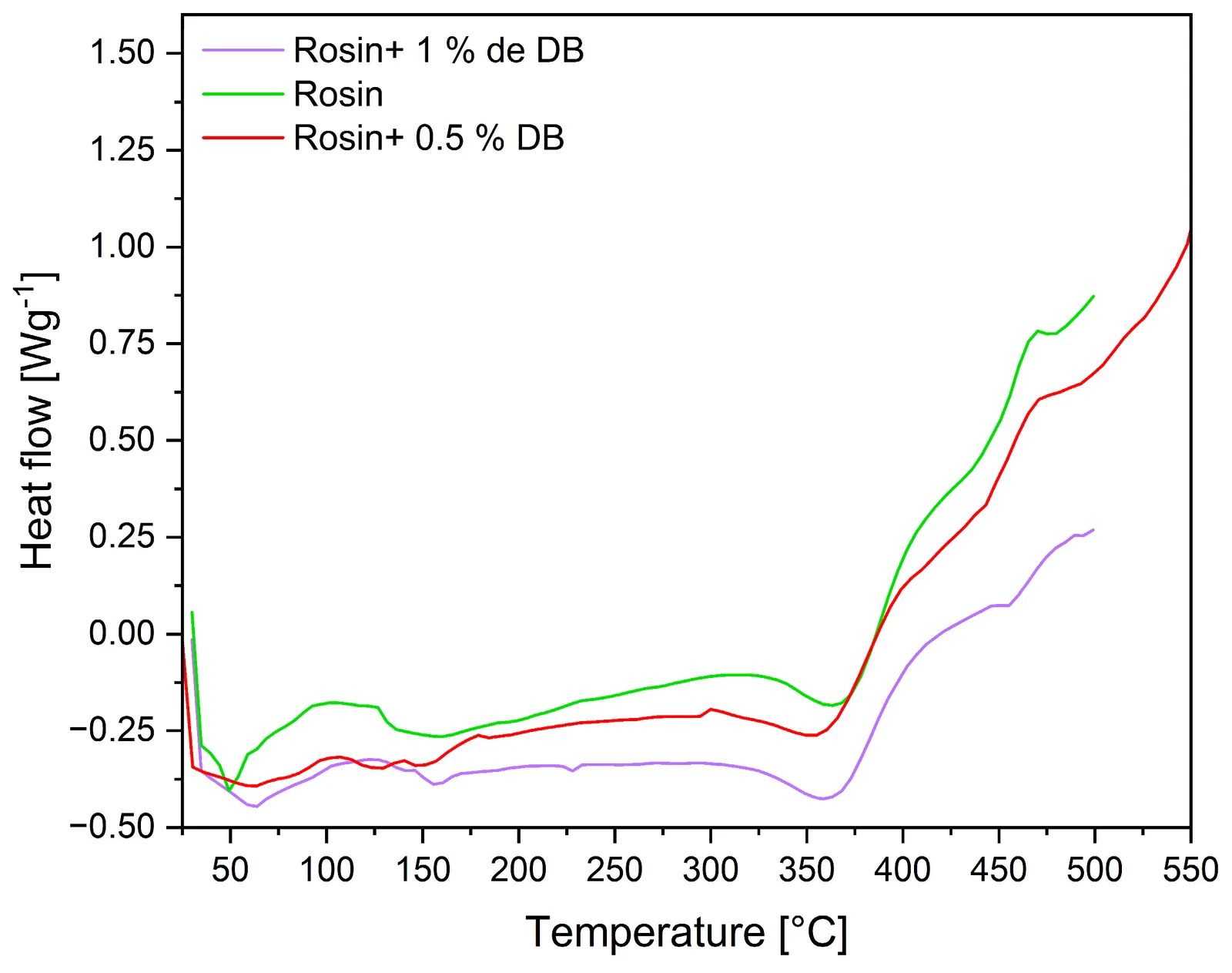
Differential scanning calorimetry (DSC) curves of the developed coatings.

The differential scanning calorimetry (DSC) profiles shown in Figure 3 exhibited a broad low-intensity endothermic event between approximately 30 and 120 °C, which has been attributed to the removal of physically adsorbed moisture together with traces of residual ethanol remaining after coating preparation [20,21].

No distinct thermal transitions, such as glass transition, melting, or crystallization peaks, were observed between 120 and 320 °C, indicating that all formulations retained the predominantly amorphous character typical of natural resin-based materials [22].

At higher temperatures, slight changes in the thermal events were observed for the formulations containing Dragon’s Blood compared with pure rosin. These differences are consistent with the improved thermal stability identified by thermogravimetric analysis and suggest that the incorporation of Dragon’s Blood did not alter the amorphous nature of the rosin matrix while contributing to enhanced thermal resistance during the degradation process. This fact has also been reported for natural resin-based materials modified with plant-derived additives, where the amorphous structure is preserved while thermal stability is slightly improved through intermolecular interactions within the polymeric matrix [23].

All analyses were performed in triplicate, yielding reproducible results. The combined FTIR, DSC, and TGA analyses demonstrated that the incorporation of Dragon’s Blood did not significantly alter the chemical structure or the predominantly amorphous character of the rosin matrix. Furthermore, the additive contributed to a slight improvement in the thermal stability of the formulations. These findings indicate that the developed coatings possessed suitable physicochemical and thermal characteristics for subsequent evaluation of their anticorrosive performance through accelerated direct-current corrosion tests and seawater immersion tests.

### 3.2. Evaluation of Anticorrosive Behavior

#### 3.2.1. Results of the Seawater Immersion Test

**Figure 4:**
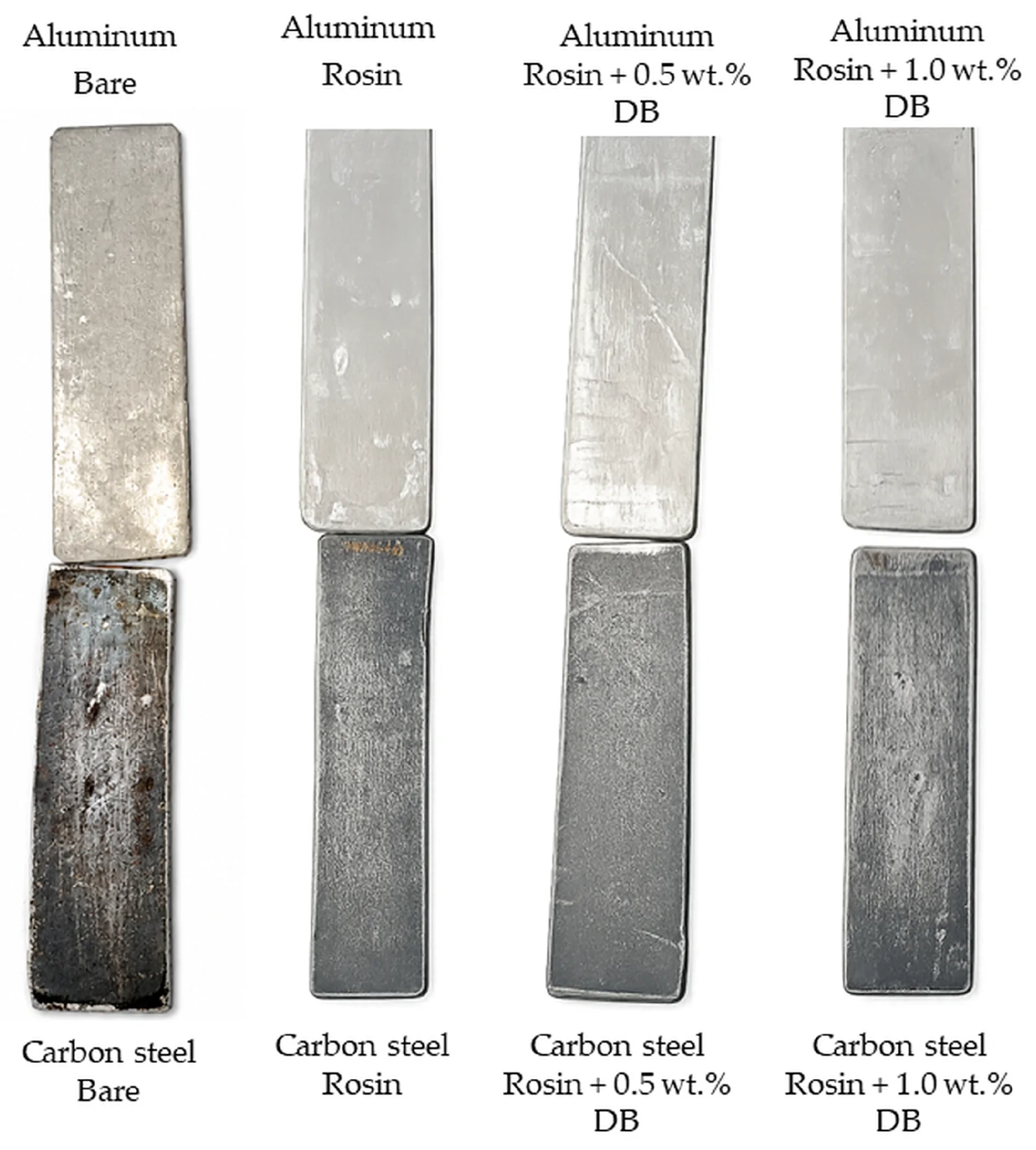
Aluminum and carbon steel specimens after 30 days of seawater immersion. From left to right: bare substrate, rosin coating, rosin coating containing 0.5 wt.% Dragon’s Blood, and rosin coating containing 1.0 wt.% Dragon’s Blood.

The anticorrosive performance of the developed coatings was evaluated through a 30-day seawater immersion test. During the exposure period, the coated face of each specimen was periodically examined to assess coating integrity, the formation of corrosion products, and surface deterioration, whereas the uncoated face served as a visual reference for corrosion progression. Representative photographs of the aluminum and carbon steel specimens after the immersion test are presented in Figure 4. These images allow visual comparison of the corrosion morphology, coating integrity, and surface deterioration observed for each coating formulation. In both substrates, the uncoated specimens exhibited more pronounced surface deterioration and localized corrosion features than the coated specimens. The rosin-based coatings reduced the extent of visible corrosion damage, with the formulation containing 0.5 wt.% Dragon’s Blood showing the best overall surface preservation, in agreement with the gravimetric corrosion results.

After completion of the test, mass loss of each specimen was determined gravimetrically.

Bare carbon steel specimens exhibited rapid formation of corrosion products throughout the immersion period, developing the characteristic reddish-brown iron oxide layer associated with severe corrosion. In contrast, the coated specimens exhibited considerably lower surface deterioration, confirming that the rosin-based coatings acted as an effective physical barrier by limiting direct contact between the metallic substrate and the corrosive medium. Although localized corrosion was observed in some coated specimens toward the end of the exposure period, the extent of degradation was substantially lower than that observed for the uncoated controls.

The gravimetric measurements summarized in Table 4 were consistent with the visual observations. For carbon steel, the uncoated specimens exhibited the highest mass loss, whereas the formulation containing rosin and 0.5 wt.% Dragon’s Blood showed the lowest mass loss, corresponding to an approximately 55.1% reduction relative to the uncoated control. Similarly, for aluminum, the mass loss decreased from 0.396 ± 0.082 g for the bare substrate to 0.106 ± 0.030 g for the formulation containing 0.5 wt.% Dragon’s Blood, representing an approximately 73.2% reduction. These results demonstrate that the incorporation of Dragon’s Blood substantially enhanced the protective performance of the rosin-based coating under prolonged seawater exposure.

The analysis of variance (ANOVA) revealed statistically significant differences among the evaluated treatments for both carbon steel (F = 18.36, *p* < 0.05) and aluminum (F = 20.15, *p* < 0.05), indicating that the coating formulation significantly affected the corrosion behavior of both metallic substrates. Tukey’s multiple comparison test identified the formulation containing 0.5 wt.% Dragon’s Blood as the best-performing treatment, exhibiting significantly lower mass loss than the remaining formulations. In contrast, the uncoated specimens consistently showed the highest degradation, whereas the rosin-only coating and the formulation containing 1.0 wt.% Dragon’s Blood displayed intermediate performance.

A consistent trend was observed for both metallic substrates, indicating that the incorporation of 0.5 wt.% Dragon’s Blood provided the most effective protective performance. On the other hand, the increase in the additive concentration to 1.0 wt.% did not provide additional corrosion protection and instead resulted in higher mass loss than the 0.5 wt.% formulation. This behavior suggests that the concentration of Dragon’s Blood reached a peak performance point within the rosin matrix. At higher concentrations, the natural additive may promote coating heterogeneity, reducing its barrier properties and facilitating electrolyte penetration through preferential pathways. Similar behavior has been reported for bio-based anticorrosive coatings containing natural additives, where excessive additive loading may compromise coating uniformity despite increasing the concentration of bioactive constituents [23].

Figure 4 illustrates the average mass loss of the coated metallic substrates after 30 days of seawater exposure. The formulation containing 0.5 wt.% Dragon’s Blood consistently exhibited the lowest mass loss for both carbon steel and aluminum, whereas increasing the additive concentration to 1.0 wt.% reduced the protective efficiency of the coating. These observations are consistent with the gravimetric measurements, the statistical analysis, and the accelerated corrosion test results presented in the following section.

Overall, the seawater immersion test demonstrated that the incorporation of 0.5 wt.% Dragon’s Blood into the rosin matrix significantly improved the anticorrosive performance of the developed coatings for both carbon steel and aluminum. The combined gravimetric measurements and statistical analyses consistently identified this formulation as the optimum treatment, highlighting the potential of Dragon’s Blood as a bio-based functional additive for the development of sustainable anticorrosive coating systems.

#### 3.2.2. Results of the Accelerated Direct-Current Corrosion Test

The anticorrosive performance of the coatings was evaluated using a DC corrosion test conducted in seawater for 60 min. Corrosion performance was assessed by comparing the mass loss of the metallic specimens after exposure. The obtained results demonstrate that the application of rosin-based coatings significantly influenced the corrosion behavior of both metallic substrates under accelerated direct-current corrosion conditions.

Table 5 summarizes the mass loss values obtained during the DC corrosion test. The uncoated carbon steel sample exhibited the highest mass loss, confirming its high susceptibility to corrosion in chloride-rich environments. During the test, rapid gas evolution and the early formation of insoluble corrosion products were observed, phenomena characteristic of accelerated corrosive attack in marine environments.

The application of rosin as the coating matrix reduced mass loss compared with the unprotected material, suggesting that the coating acts as a physical barrier that limits electrolyte diffusion toward the metallic surface. However, the presence of corrosion products indicates that the protective film is not completely impermeable, allowing the gradual penetration of aggressive species through microdefects or regions of lower continuity.

The incorporation of Dragon’s Blood (*Croton lechleri*) significantly improved the corrosion behavior of the coating system under accelerated direct-current corrosion conditions. In particular, the formulation containing 0.5 wt.% Dragon’s Blood exhibited the lowest mass loss in both materials, resulting in a substantial reduction compared with the unprotected samples. This result suggests that the incorporation of Dragon’s Blood promoted the formation of a more compact and stable protective film, improving the barrier properties of the coating and reducing substrate deterioration.

In contrast, the formulation containing 1.0 wt.% Dragon’s Blood did not provide additional improvement in protective performance. In carbon steel, mass loss reached values similar to, and even slightly higher than, those observed for the blank sample. This behavior may be associated with the formation of heterogeneities, microvoids, or discontinuities within the coating when higher concentrations of the additive are employed, facilitating electrolyte penetration and promoting localized corrosion processes.

ANOVA revealed statistically significant differences among the evaluated treatments for both carbon steel (F = 55.87, *p* < 0.05) and aluminum (F = 14.86, *p* < 0.05). Tukey’s multiple comparison test consistently identified the formulation containing 0.5 wt.% Dragon’s Blood as the best-performing treatment, exhibiting significantly lower mass loss than the remaining formulations. In contrast, the uncoated specimens showed the highest deterioration, whereas the rosin-only coating and the formulation containing 1.0 wt.% Dragon’s Blood displayed intermediate performance.

**Figure 5:**
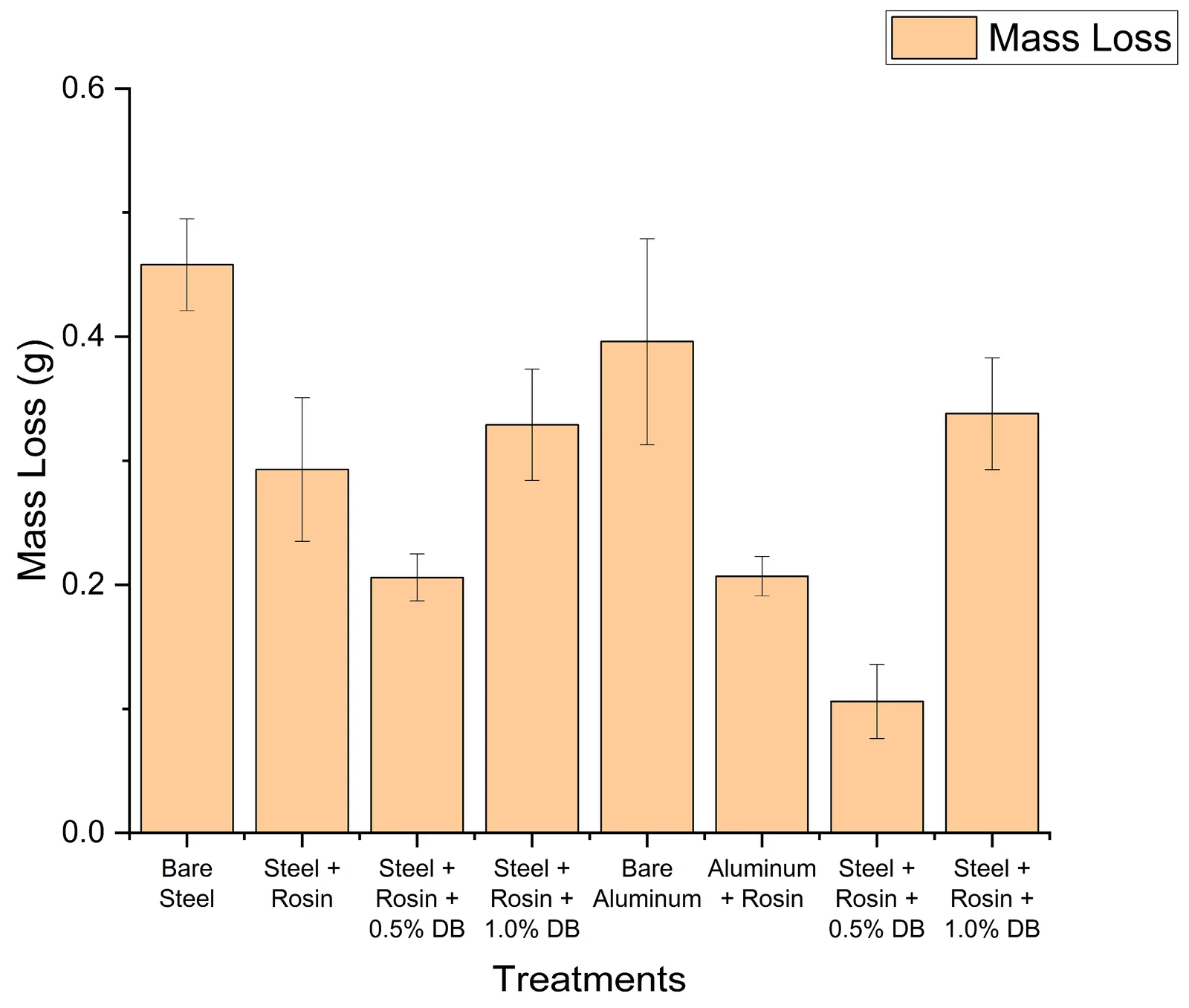
Comparison of the average mass loss of coated samples after 30 days of exposure to seawater.

As shown in Figure 5, both materials exhibited a decrease in mass loss when the treatments were applied up to the rosin + 0.5 wt.% Dragon’s Blood condition. However, in the formulation containing 1.0 wt.% of the additive, the behavior differed between materials, with a more pronounced increase observed in carbon steel than in aluminum.

**Figure 6:**
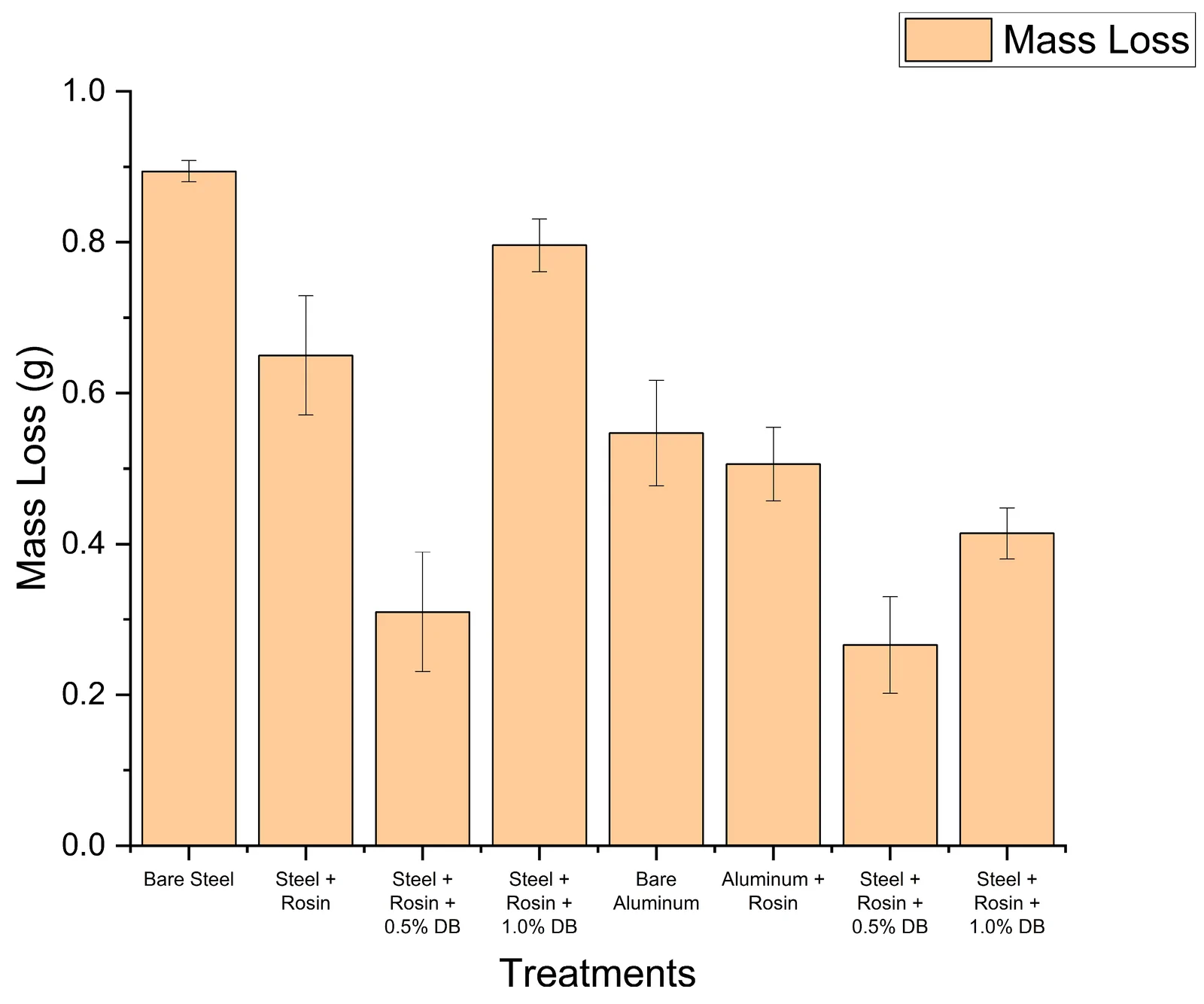
Comparison of the average mass loss of coated samples in the accelerated direct-current corrosion test.

The differences observed between the accelerated direct-current corrosion test and immersion tests are expected due to the fact that the accelerated DC corrosion method accelerates corrosion by imposing an external electrical potential, whereas the immersion test evaluates the long-term barrier performance of the coating under natural exposure conditions. Consequently, the accelerated DC corrosion test is more sensitive to localized coating defects and early electrolyte penetration, whereas the seawater immersion test provides a more representative assessment of the long-term barrier performance of the developed coatings. Figure 6 summarizes the mass loss results obtained for the different coating formulations after the accelerated direct-current corrosion test.

Overall, the accelerated DC corrosion test demonstrated that the anticorrosive performance of the coatings depended on the concentration of Dragon’s Blood incorporated into the rosin matrix. The formulation containing 0.5 wt.% Dragon’s Blood consistently exhibited the lowest mass loss for both metallic substrates, suggesting improved barrier properties and greater resistance to electrolyte penetration. Although this accelerated test is more sensitive to localized coating defects and accelerates the corrosion process compared with the seawater immersion test, both evaluation methods consistently identified the formulation containing 0.5 wt.% Dragon’s Blood as the optimum coating system, confirming its potential as a sustainable bio-based anticorrosive coating for metallic substrates exposed to marine environments.

## 4. Discussion

The superior anticorrosive performance observed for the coating containing 0.5 wt.% Dragon’s Blood in both the seawater immersion test and the accelerated DC corrosion test suggests that a maxim level of additive efficacy is reached at this concentration, where Dragon’s Blood is most effectively incorporated into the rosin matrix.

At this concentration, the bioactive constituents naturally present in Dragon’s Blood, particularly its phenolic compounds, may contribute to improving the barrier properties of the coating by promoting a more homogeneous distribution within the rosin matrix and reducing the diffusion of aggressive species such as water and chloride ions [12]. This interpretation is consistent with the thermal and physicochemical characterization results, which indicated adequate stability of the developed formulations.

ATR-FTIR analyses suggested that the incorporation of Dragon’s Blood did not significantly modify the chemical structure of the rosin matrix, whereas DSC demonstrated that the predominantly amorphous character of the formulations was preserved. In addition, TGA revealed a slight improvement in thermal stability, suggesting that the enhanced anticorrosive performance resulted primarily from improved physical barrier properties rather than from chemical modification of the resin matrix [24].

In contrast, increasing the Dragon’s Blood content to 1.0% did not result in additional corrosion protection. This fact suggests that higher concentrations of the natural extract may lead to the formation of localized domains or agglomerates within the polymeric matrix, generating structural heterogeneities that facilitate the penetration of the corrosive medium [25,26]. Furthermore, Dragon’s Blood is rich in polyphenolic compounds containing hydroxyl groups, which may increase the affinity of the coating for water when incorporated in excessive amounts [13]. Consequently, although these compounds can contribute to corrosion inhibition, their excessive presence may partially compromise the barrier effect of the coating [14].

A similar behavior has been reported for other bio-based additives incorporated into polymeric systems, where the best performance is frequently achieved at intermediate concentrations rather than at the highest additive loading [27]. Therefore, the results obtained in this study suggest that the protective effect of Dragon’s Blood is concentration-dependent, with 0.5 wt.% representing the most favorable balance between additive incorporation, coating integrity, and corrosion resistance. Overall, the combined physicochemical, thermal, and corrosion results demonstrate that the protective efficiency of the developed coatings depends on both the presence of Dragon’s Blood and its effective incorporation within the rosin matrix. Therefore, increasing the additive concentration beyond the optimum level does not necessarily improve coating performance and may even reduce its protective effectiveness by affecting coating homogeneity [28].

## 5. Conclusions

The present study demonstrated that rosin-based coatings modified with Dragon’s Blood (*Croton lechleri*) can be successfully developed as bio-based anticorrosive coatings. ATR-FTIR analyses confirmed that the incorporation of Dragon’s Blood did not significantly alter the chemical structure of the rosin matrix, whereas DSC and TGA analyses indicated that the developed formulations maintained adequate thermal stability for coating applications.

Among the evaluated formulations, the coating containing 0.5 wt.% Dragon’s Blood exhibited the best anticorrosive performance for both carbon steel and aluminum, producing reductions in mass loss of approximately 55.1% and 73.2%, respectively, during the 30-day seawater immersion test.

Both the seawater immersion test and the accelerated DC corrosion test consistently identified the formulation containing 0.5 wt.% Dragon’s Blood as the optimum coating system. The statistical analyses confirmed significant differences among treatments, demonstrating that moderate incorporation of the natural additive improved the barrier properties of the rosin matrix and reduced substrate deterioration under corrosive conditions.

In contrast, the increase in the Dragon’s Blood content to 1.0 wt.% did not provide additional protection and, in some cases, resulted in lower protective performance. This behavior suggests that excessive additive loading may reduce coating homogeneity and consequently decrease its barrier properties.

Overall, the results demonstrate the potential of Dragon’s Blood as a sustainable bio-based functional additive for rosin-based coatings. The combination of physicochemical characterization, thermal analyses, and corrosion testing confirmed that the formulation containing 0.5 wt.% Dragon’s Blood provided the best balance between coating integrity, thermal stability, and anticorrosive performance. These findings support the development of environmentally friendly protective coatings for metallic substrates exposed to aggressive marine environments.

## Figures and Tables

**Table 1:** Experimental design and distribution of coated specimens according to substrate, coating formulation, and corrosion test.

Substrate Material	Coating Formulation	Seawater Immersion Test (n)	Accelerated DC Corrosion Test (n)	Total Specimens
Carbon steel	Blank	3	3	6
	Rosin	3	3	6
	Rosin + 0.5 wt.% DB	3	3	6
	Rosin + 1.0 wt.% DB	3	3	6
Subtotal		12	12	24
Aluminum	Blank	3	3	6
	Rosin	3	3	6
	F Rosin + 0.5 wt.% DB 2	3	3	6
	Rosin + 1.0 wt.% DB	3	3	6
Subtotal		12	12	24
Total		24	24	48

**Table 2:** Formulations of the evaluated natural coatings.

Formulation	Rosin (g)	Dragon’s Blood Resin (g)	Dragon’s Blood Resin Concentration (wt.% Relative to Rosin)
Rosin	10.00	0.00	0.0
Rosin + 0.5 wt.% DB	10.00	0.05	0.5
Rosin + 1.0 wt.% DB	10.00	0.10	1.0

**Table 3:** Characteristic thermal degradation parameters obtained from TGA.

Formulation	T5% (°C)	T10% (°C)	Tmax (°C)
Rosin	199.8	220.4	269.6
Rosin + 0.5 wt.% DB	210.3	234.9	299.8
Rosin + 1.0 wt.% DB	195.8	223.8	299.9

**Table 4:** Mass loss of metallic samples after 30 days of seawater immersion.

Material	Coating System	Mass Loss (g, Mean ± SD)
Carbon steel	Bare	0.458 ± 0.037
	Rosin	0.293 ± 0.058 bc
	Rosin + 0.5 wt.% DB	0.206 ± 0.019 c
	Rosin + 1.0 wt.% DB	0.329 ± 0.050 b
Aluminum	Bare	0.396 ± 0.082 a
	Rosin	0.207 ± 0.016 b
	Rosin + 0.5 wt.% DB	0.106 ± 0.030 c
	Rosin + 1.0 wt.% DB	0.338 ± 0.045 a

Values are expressed as mean ± standard deviation (*n* = 3). Different lowercase letters within each metallic substrate indicate significant differences according to Tukey’s multiple comparison test (*p* < 0.05).

**Table 5:** Average mass loss of metallic samples in the accelerated direct-current corrosion test.

Material	Coating System	Mass Loss (g)
Carbon steel	Bare	0.854 ± 0.024 a
	Rosin	0.613 ± 0.021 bc
	Rosin + 0.5 wt.% DB	0.333 ± 0.040 c
	Rosin + 1.0 wt.% DB	0.865 ± 0.037 a
Aluminum	Bare	0.312 ± 0.007 a
	Rosin	0.463 ± 0.033 a
	Rosin + 0.5 wt.% DB	0.237 ± 0.011 b
	Rosin + 1.0 wt.% DB	0.372 ± 0.016 ab

Values are expressed as mean ± standard deviation (*n* = 3). Different letters within each material indicate significant differences according to Tukey’s test (*p* < 0.05).

## Data Availability

The original contributions presented in this study are included in the article. Further inquiries can be directed to the corresponding author.

## Abbreviations

The following abbreviations are used in this manuscript:

DB	Dragon’s Blood
DC	Direct Current
FTIR	Fourier Transform Infrared Spectroscopy
TGA	Differential Scanning Calorimetry
DCS	Thermogravimetric Analysis

## References

[1] Bastidas D.M. (2020). Corrosion and Protection of Metals.

[2] Ionescu G.C., Ionescu G.L., Badea G.E., Marin L. (2024). Modern Theoretical and Practical Protection Methods of Metallic Structures against the Effects of Corrosion. J. Appl. Eng. Sci..

[3] Yan Z., Shu S., Yang Y., Du B. (2025). Surface and Electrochemical Performance Variations of 2205 Duplex Stainless Steel in Multistep Surface Processing. ACS Omega.

[4] Pavón Vargas C. (2022). Aditivación de Materiales Biodegradables Mediante El Uso de Derivados de Colofonia. Ph.D. Thesis.

[5] Cuevas-Suárez C., Ortiz-Rodríguez L., Alonso-Hernández C. (2024). Obtención y Rendimiento de Colofonia a Partir de Resina de Pinus Teocote. Cienc. Lat. Rev. Científica Multidiscip..

[6] Valderrama Celestino C. (2024). Síntesis y Caracterización de Un Recubrimiento Compuesto Polímero—Nanoestructuras Para Evaluar Sus Propiedades Antibioincrustantes. Master’s Thesis.

[7] Caclamanis J.P. (2024). Efectividad de Un Recubrimiento Polimérico Autorreparable Sobre Acero. Master’s Thesis.

[8] Cabezas J. (2020). Elaboración y Caracterización Física de Recubrimientos Biodegradables Obtenidos a Partir de Cáscara y Semilla de Sandía (*Citrullus lanatus*). Bachelor’s Thesis.

[9] Carrión-Mero P., Montalván F.J., Morante-Carballo F., Heredia J., Elorza F.J., Solórzano J., Aguilera H. (2021). Hydrochemical and Isotopic Characterization of the Waters of the Manglaralto River Basin (Ecuador) to Contribute to the Management of the Coastal Aquifer. Water.

[10] Bogatu N., Buruiana D.L., Muresan A.C., Ghisman V., Lupu A., Mardare L., Herbei E.E., Basliu V., Ceoromila A., Florescu S. (2025). Assessment of the Effectiveness of Protective Coatings in Preventing Steel Corrosion in the Marine Environment. Polymers.

[11] Thai N.X., Chinh N.T., Quan V.A., Hung D.P., Hiep N.A., Dung H.T., Khanh H.D., Hoang T. (2023). Assessment of Marine Antifouling Property of Fluoropolymer Nanocomposite Coating on Steel Substrate—A Preliminary Assay in Laboratory. Vietnam J. Chem..

[12] Cevallos-Morillo C., Cisneros-Pérez P., Llive R., Ricaurte M., Reinoso C., Meneses M.A., Guamán M.D.C., Palma-Cando A. (2021). Croton Lechleri Extracts as Green Corrosion Inhibitors of Admiralty Brass in Hydrochloric Acid. Molecules.

[13] Ambarita A.C., Mulyati S., Arahman N., Bilad M.R. (2024). Characterization of Dragon Blood Resins and Their Potential as Modifying Agents for Hydrophobic Membranes. Int. J. Des. Nat. Ecodynamics.

[14] Taghavikish M., Dutta N.K., Choudhury N.R. (2017). Emerging Corrosion Inhibitors for Interfacial Coating. Coatings.

[15] Yu S., Cao J., Li S., Huang H., Li X. (2023). The Tribological and Mechanical Properties of PI/PAI/EP Polymer Coating under Oil Lubrication, Seawater Corrosion and Dry Sliding Wear. Polymers.

[16] Escobar J.D., Prieto C., Pardo-Figuerez M., Lagaron J.M. (2018). Dragon’s Blood Sap: Storage Stability and Antioxidant Activity. Molecules.

[17] Wongsa P., Phatikulrungsun P., Prathumthong S. (2022). FT-IR Characteristics, Phenolic Profiles and Inhibitory Potential against Digestive Enzymes of 25 Herbal Infusions. Sci. Rep..

[18] Hoque M., Alam M., Wang S., Zaman J.U., Rahman M.S., Johir M.A.H., Tian L., Choi J.G., Ahmed M.B., Yoon M.H. (2023). Interaction Chemistry of Functional Groups for Natural Biopolymer-Based Hydrogel Design. Mater. Sci. Eng. R Rep..

[19] Brito J., Hlushko H., Abbott A., Aliakseyeu A., Hlushko R., Sukhishvili S.A. (2021). Integrating Antioxidant Functionality into Polymer Materials: Fundamentals, Strategies, and Applications. ACS Appl. Mater. Interfaces.

[20] Olejnik O., Masek A., Kiersnowski A. (2020). Thermal Analysis of Aliphatic Polyester Blends with Natural Antioxidants. Polymers.

[21] Aldas M., Pavon C., López-Martínez J., Arrieta M.P. (2020). Pine Resin Derivatives as Sustainable Additives to Improve the Mechanical and Thermal Properties of Injected Moulded Thermoplastic Starch. Appl. Sci..

[22] Escobar-Avello D., Oñate-Valdés T., Ferrer V., Fuentealba C., Benavides-Valenzuela S., Cabrera-Barjas G., Bravo-Arrepol G., Giordano A., Gullón B., Santos J. (2026). Optimization of Emerging Extraction Techniques for Phenolic Compounds from Pinus Radiata Bark: Antioxidant, Thermal Stability and Antibacterial Properties. Antioxidants.

[23] Chang C.W., Lee J.J., Lu K.T. (2021). The Effects of Adding Heartwood Extractives from Acacia Confusa on the Lightfastness Improvement of Refined Oriental Lacquer. Polymers.

[24] Fang H., Dai Y., Lu Z., Yang Z., Wei Y. (2023). Enhancement of Barrier and Corrosion Protection Performance of Vinyl Ester Resin Coating via Incorporation of MXene Nanosheets. Results Eng..

[25] Lahbabi S., Bouferra R., Saadi L., Khalil A. (2025). Evaluation of Tamarix Aphylla Extract as an Eco-Friendly Corrosion Inhibitor for Carbon Steel in Saline Medium. RSC Adv..

[26] Golshani Z., Arjmand F., Amiri M., Mohammad S., Hosseini A., Fatemi S.J. (2023). Investigation of Dracocephalum Extract Based on Bulk and Nanometer Size as Green Corrosion Inhibitor for Mild Steel in Different Corrosive Media. Sci. Rep..

[27] Dobrotă D., Bărbușiu A.M., Sava G.A., Oleksik V. (2025). Ștefan Functional Additives in Automotive Polymer Matrices: Compatibility, Mechanisms, and Industry Challenges. Polymers.

[28] Ambarita A.C., Arahman N., Bilad M.R., Gül B.Y., Korkut S., Yüksekdağ A., Teber O.O., Koyuncu İ., Mulyati S. (2023). Polyethersulfone Membrane Coated with Dragon Blood Resin: Effect of Input Parameters and Optimization Using Response Surface Methodology. S. Afr. J. Chem. Eng..

